# Lentinan improved the efficacy of vaccine against *Trichinella spiralis* in an NLRP3 dependent manner

**DOI:** 10.1371/journal.pntd.0008632

**Published:** 2020-09-25

**Authors:** Xuemin Jin, Xiaolei Liu, Jing Ding, Lixiao Zhang, Yaming Yang, Xuelin Wang, Yong Yang, Mingyuan Liu

**Affiliations:** 1 Key Laboratory of Zoonosis Research, Ministry of Education, Institute of Zoonosis, College of Veterinary Medicine, Jilin University, Changchun, China; 2 Yunnan Institute of Parasitic Diseases, Puer, Yunnan, China; 3 Jiangsu Co-innovation Center for Prevention and Control of Important Animal Infectious Diseases and Zoonoses, Yangzhou, Jiangsu, PR China; Istituto Superiore di Sanità, ITALY

## Abstract

There is an urgent need for the development of new, improved vaccine adjuvants against *T*. *spiralis* infection. Polysaccharides are effective, safe, and biodegradable as adjuvant. In our study, we first observed the protective efficacy of lentinan as adjuvant against helminth *T*. *spiralis* infection. Recombinant *T*. *spiralis* Serpin (r*Ts*-Serpin) immunoscreened from a cDNA library of *T*. *spiralis*, as a vaccine, protect host against *Trichinella* infection. The reduction rate of helminth burden of r*Ts*-Serpin+lentinan–immunized mice was significantly increased compared with r*Ts*-Serpin+FCA -immunized mice. r*Ts*-Serpin+lentinan induced IgG1-dominant immune response and higher levels of IFN-γ and IL-4. r*Ts*-Serpin+lentinan displayed a lower reduction rate of parasite burden in NLRP3^-/-^ mice than that in WT mice and lower level of IgG1 than that in WT mice. The level of IL-4, but not IFN-γ, from NLRP3^-/-^ mice immunized by r*Ts*-Serpin+lentinan was significantly lower than that from WT mice, suggesting that NLRP3 is associated with r*Ts*-Serpin+lentinan -triggering Th2 protective immunity against *T*. *spiralis* infection. In summary, we revealed that lentinan was a novel adjuvant against *T*. *spiralis* infection via NLRP3. NLRP3 therefore represents an important target for adjuvant discovery and the control of *T*. *spiralis* infection.

## Author summary

*Trichinella spp*., pathogenic agents of trichinellosis, is foodborne zoonotic nematodes cause huge economic burden to the livestock industry. The potential of new adjuvants for improving veterinary vaccines remains largely unexploited to trigger safe and long-lasting immunity in large animals, including livestock. Polysaccharides are effective, safe, and biodegradable as adjuvant. We first observed the protective efficacy of lentinan as a novel adjuvant against helminth *T*. *spiralis* infection. NLRP3 is associated with lentinan -triggering Th2 protective immunity against *T*. *spiralis* infection. NLRP3 therefore represents an important target for adjuvant discovery and the control of *T*. *spiralis* infection.

## Introduction

Parasitic diseases are a serious global health concern. Trichinellosis, caused by *Trichinella spiralis* (*T*. *spiralis*), is one of the most prevalent neglected tropical diseases worldwide, and establishes chronic infection in a wide range of wild and domestic animals and human beings [1]. Few studies concern the protective immunity of adjuvant against *T*. *spiralis* infection [2]. Most of vaccination trials have been performed in mice or pigs generally with Freund complete adjuvant (FCA) [2]. However, due to the toxicity of FCA, its use is unacceptable, which leads to animal pain and damage to meat quality. The new-generation adjuvants of water-in oil emulsions (w/o) after being mixed with antigens, which have favorable adjuvant characteristics for eliciting a long-term and strong immune response [3], however, they can cause local and systemic reactions such as granulomas, abscesses or fever [4]. Aluminium-based adjuvants have the simplicity and tolerability, however, high aluminum levels lead to reduce renal function, may affect the brain and bone tissues resulting in neurological syndrome and dialysis-associated dementia [5]. Therefore, there is an urgent need for the development of new, improved vaccine adjuvants against *T*. *spiralis* infection.

Many polysaccharides from plant, bacterial, yeast and synthetic sources can act as pathogen-associated molecular patterns (PAMPs) and recognize pattern recognition receptors (PRRs) on immune cells, followed by regulating immunity [6–9]. Polysaccharides are safe, and biodegradable, with no tissue deposition [10]. Lentinan is purified β-glucan from Shiitake mushrooms. Lentinan has been approved as a biological response modifier for cancer [11] and as adjuvants for virus disease [12]. Glucans were found to be the most promising vaccine adjuvant, as they alone stimulate the immune system including antibody production without any side effects [13, 14]. However, the efficiency of lentinan as adjuvant against helminth infection such as *T*. *spiralis* is still unknown.

*T*. *spiralis* infection induces strong T helper 2 (Th2) immune response [15], which contributes equally to host defense against *T*. *spiralis* [16]. IL‐4, a Th2-related cytokine played a role in the expulsion of *T*. *spiralis* in the host [17]. Notably, transcription factor NLRP3 (nod-like receptor (NLR) family, pyrin domain containing 3) in CD4+ T cells acts as a key transcription factor in Th2 immune response [18], which is involved in protective immunity to helminth infection. It has been reported that NLRP3 activation is essential for the control of different parasitic infections. Activation of the NLRP3 inflammasome reduces *Toxoplasma gondii* infection load [19] and is critical for host resistance to diverse *Leishmania* species [20]. Furthermore, NLRP3 contributes to adjuvanticity *in vivo* [21]. However, it is unclear whether NLRP3 is activated and involved in protective immunity against *T*. *spiralis* infection.

Previously, we demonstrated that an antigenic protein, *Ts*-Serpin immunoscreened from a cDNA library of *T*. *spiralis* [22], as a vaccine, protect the host against *Trichinella* infection [23]. In this study, we found that lentinan as an adjuvant improved the protective efficacy of this vaccine against *T*. *spiralis*. We revealed the critical role of NLRP3 in promoting Th2 responses through lentinan based on vaccine, indicating that NLRP3 represents an important target to screen more adjuvant for the control of *T*. *spiralis* infection.

## Materials and methods

### Ethics statement

C57BL/6J wild-type (WT) mice (female, 4–6 weeks old) were purchased from the Norman Bethune University of Medical Science (NBUMS), China. Female Wistar rats were purchased from the Experimental Animal Centre of College of Basic Medical Sciences, Jilin University (Changchun, China). C57BL/6J NLRP3^-/-^ mice were kindly provided by Dr. Feng Shao. All animals were maintained on standard rodent chow with water supplied *ad libitum* under a 12 h/12 h light/dark cycle during the experimental period. All animal experiments were performed according to the regulations of the Administration of Affairs Concerning Experimental Animals in China. The protocol was approved by the Institutional Animal Care and Use Committee of Jilin University (20170318).

### T. spiralis

The *T*. spiralis isolate (ISS534) was obtained from a naturally infected domestic pig in Henan Province in China and maintained in rats [24]. Briefly, Wistar rats were orally infected with 3000 infective larvae, and *T*. *spiralis* muscle larvae were recovered at 35 days post infection (dpi) *via* artificial digestion with pepsin-HCl (1% pepsin and 1% HCl at 37°C for 2 h) [25].

### Preparation of recombinant *Ts*-Serpin (r*Ts*-Serpin)

Recombinant *Ts*-Serpin (r*Ts*-Serpin) was expressed in *E*. *coli (BL21)* and purified as previously described [23]. The contaminated endotoxin was effectively removed by ToxOut High Capacity Endotoxin Removal Kit (Biovision, USA). The residual endotoxin was 0.1412 EU/mg in the final purified r*Ts*-Serpin, approximately equivalent to 20 pg/mg endotoxin in r*Ts*-Serpin [26].

### Formulation of r*Ts*-Serpin with adjuvants

Complete and incomplete Freund’s adjuvants (FCA/FIA) were purchased from Sigma (St. Louis, Mo, USA). FCA/FIA formulations were prepared by emulsifying equal volumes of adjuvant with r*Ts*-Serpin. Lentinan (Medchemexpress, China) (10 mg/kg) were resuspended in PBS and r*Ts*-Serpin (50μg per mice) was dropped to a final ratio of 1:1 (v/v) slowly, with gentle shaking of the r*Ts*-Serpin solution.

### Immunization and challenge infection

To determine the protective potential of the lentinan, female C57BL/6J mice were randomly divided into 4 groups of 20 mice each: (1) immunized subcutaneously with PBS only, as control, (2) immunized subcutaneously with 50 μg of r*Ts*-Serpin, (3) immunized subcutaneously with 50 μg of r*Ts*-Serpin emulsified with Freund’s adjuvants (FCA/FIA), (4) immunized subcutaneously with r*Ts*-Serpin emulsified with lentinan. Mice were subcutaneously immunized in different site. Immunization was performed 3 times using the same method at 2-week interval. Two weeks after the final vaccination, all mice were orally challenged with 500 *T*. *spiralis* muscle larvae.

### Parasite burden assessments

Intestinal adult worms were collected at 7 dpi, and muscle larvae were recovered and counted at 35 dpi as previously described [27]. The protective immunity was calculated based on the percent of reduction in the mean number of adult worms or the recovered muscle larvae per gram (LPG) of muscle from vaccinated groups compared with PBS group.

### Antibody assays

Specific antibodies against r*Ts*-Serpin were assayed at 7 weeks post vaccination. Blood was collected from vaccinated mice at 2, 4, 6 and 7 wpv. The sera samples were at a 1:100 dilution. The titers of anti- r*Ts*-Serpin IgG, IgG1, IgG2a subclasses, and IgE was measured using an indirect enzyme-linked immunosorbent assay (ELISA) as described previously [28]**.**

### Cytokine assays

To examine Th1 and Th2 response in splenocytes from immunized mice upon stimulation with the r*Ts*-Serpin antigen, the cytokine profile from splenocyte culture supernatants was tested. Briefly, 1 week after the final immunization, CD4^+^ T cells from mice were purified from spleen cells by magnetic sorting using anti-CD4 magnetic beads (Miltenyi Biotec, Auburn, CA). The purified CD4^+^ T cells had > 90% purity. The CD4^+^ T cells were modulated to 1 × 10^6^ cells/mL in complete RPMI-1640 with 10% fetal bovine serum (FBS), penicillin (100 U/mL) and streptomycin (100 μg/mL) and stimulated with r*Ts*-Serpin at a concentration of 20 μg/mL at 37°C for 72 hours in an incubator containing 5% CO_2_. The supernatants of CD4^+^ T cells were harvested. IFN-γ and IL-4 levels in the supernatant were quantified by ELISA (R&D Systems).

### qRT-PCR

Total RNA was extracted from CD4^+^ T cells using TRIzol reagent (Invitrogen, Carlsbad, CA, USA) according to the manufacturer’s instructions. RNA was reverse transcribed into cDNA using random primers from a TransScript One-Step gDNA removal kit and cDNA synthesis supermix (TransGen Biotech, China). The mRNA expression of NLRP3 was quantified by a SYBR-Green fluorescent dye detection system (Roche Applied Science) on an ABI 7500 Fast Real-Time PCR System using the appropriate forward and reverse primers, which are listed in Table 1 [29]. PCR conditions were as follows: 95°C for 10 min, followed by 45 cycles of amplification at 95°C for 15 s and 60°C for 60 s. Relative gene expression was normalized to the housekeeping control gene (GAPDH) using the 2^-ΔΔCT^ method [30].

**Table 1:** The primers of quantitative RT-PCR.

Genes	Primer	Sequence(5′→3′)
**NLRP3**	Forward primer	TGTGTGGATCTTTGCTGCG
	Reverse primer	GTTACTGTGCACATGTAGTGTAAGG‬‬‬‬‬‬‬‬‬‬‬‬‬‬‬‬‬‬
**GAPDH**	Forward primer	ACTCCACTCACGGCAAATTC
	Reverse primer	TCTCCATGGTGGTGAAGACA

### Statistical analysis

All results are expressed as the mean ± SD. Statistical analysis was performed using GraphPad Prism 8 for Windows. Two experimental groups were compared using Student’s t-test for nonparametric data. Three or more groups were compared through one-way analysis of variance (ANOVA) with Tukey's multiple comparison test or Dunnett’s multiple comparison test as indicated. P values are expressed as *p<0.05, **p< 0.01 and ***p<0.001. The numerical data used in all figures are included in S1 Data.

## Result

### Lentinan improved the protective efficacy of vaccination of r*Ts*-Serpin against *T*. *spiralis* infection

**Fig 1:**
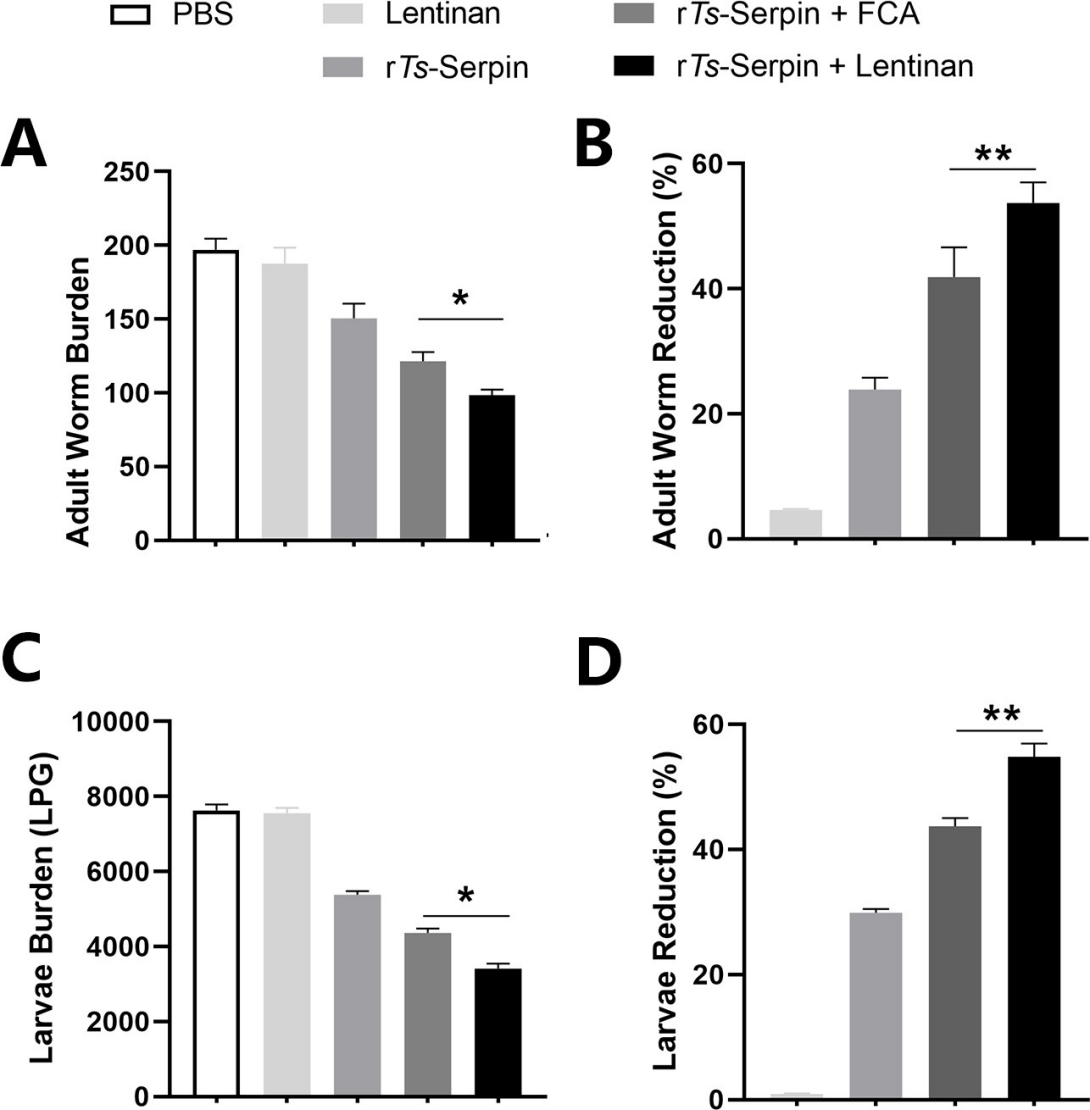
Protective immunity induced by immunizing mice with lentinan against *T*. *spiralis*. The number of adults recovered from intestines (A) and muscle larvae (ML) per gram (LPG) in skeletal muscles (B) from mouse groups immunized subcutaneously with PBS (PBS), r*Ts*-Serpin (r*Ts*-Serpin), r*Ts*-Serpin emulsified with Freund’s adjuvants (r*Ts*-Serpin+FCA, and r*Ts*-Serpin emulsified with lentinan (r*Ts*-Serpin+lentinan), after challenge with 500 ML of *T*. *spiralis*. The adult worms (C) and muscle larvae (D) reduction rates were analyzed based on the the mean number of adult worms or the recovered muscle larvae per gram (LPG) of muscle from vaccinated groups compared with PBS group. Results are expressed as the mean ± SD of 10 mice per group. The data shown are representative of three independent experiments. *p<0.05, ** p < 0.01, vs the as indicated by the line (Tukey multiple comparison following ANOVA).

To evaluate the protective efficacy of lentinan as adjuvant against *T*. *spiralis* infection, lentinan combined with r*Ts*-Serpin was immunized prior to *T*. *spiralis* infection. Our results showed that immunization of lentinan only failed to significantly reduce the burden of AW and ML. r*Ts*-Serpin significantly reduced the burden of *T*.*spiralis* compared to PBS group (Fig 1A and 1C). Compared with mice in PBS or r*Ts*-Serpin group, mice in r*Ts*-Serpin + Freund’s adjuvants (FCA) group, r*Ts*-Serpin+lentinan group displayed lower adult worm burden and muscle larvae burden. And r*Ts*-Serpin + lentinan significantly reduced the parasite burden compared with r*Ts*-Serpin + FCA (Fig 1A and 1C). The reduction rate of r*Ts*-Serpin + lentinan (AW: 53.71%, p<0.01; ML: 54.83%, p<0.01) was significantly increased compared with r*Ts*-Serpin + FCA (AW: 41.87%, ML: 43.75%) (Fig 1B and 1D).

### r*Ts*-Serpin+lentinan elevated the levels of specific immunoglobulin responses and Th1/Th2 cytokines

**Fig 2:**
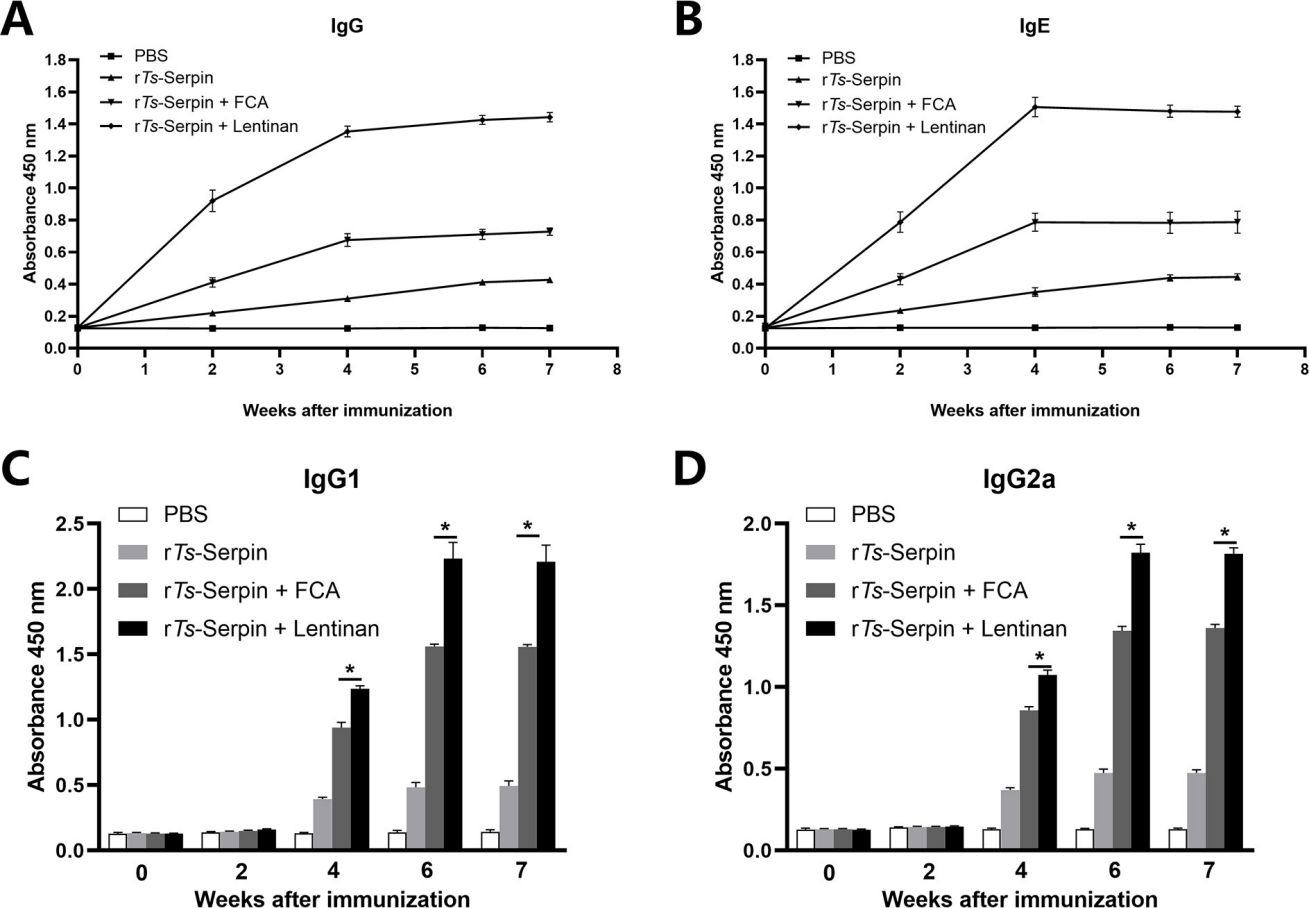
Analysis of humoral immune responses. (A) The levels of anti-r*Ts*-serpin IgG in the sera of immunized mice or control mice were measured by ELISA. (B) The levels of specific IgE r*Ts*-serpin in the sera of immunized mice were measured. (C) The IgG1 subclass responses against r*Ts*-serpin were detected at different time points. (D) The IgG2a subclass responses against r*Ts*-serpin were detected at different time points. The values shown for each group are the mean + SD of the antibody levels (n = 10) from three individual experiments * p < 0.05 as indicated by the line (one-way ANOVA with Tukey’s post-test).

The results showed significant increase in total IgG level in mice immunized with r*Ts*-Serpin + lentinan compared with r*Ts*-Serpin + FCA group after the second immunization (Fig 2A). The IgG subtype assay indicated that IgG1 and IgG2a responses were both significantly increased in mice immunized by r*Ts*-Serpin + lentinan, compared with mice immunized by r*Ts*-Serpin + FCA (Fig 2C and 2D). The IgG1 level was higher than IgG2a level, indicating that immunization with r*Ts*-Serpin + lentinan induced IgG1-dominant Th1 (IgG2a)/Th2 (IgG1) -mixed immune response. And the levels of specific IgE were also significantly increased in mice immunized with r*Ts*-Serpin + lentinan compared with the r*Ts*-Serpin + FCA (Fig 2B).

**Fig 3:**
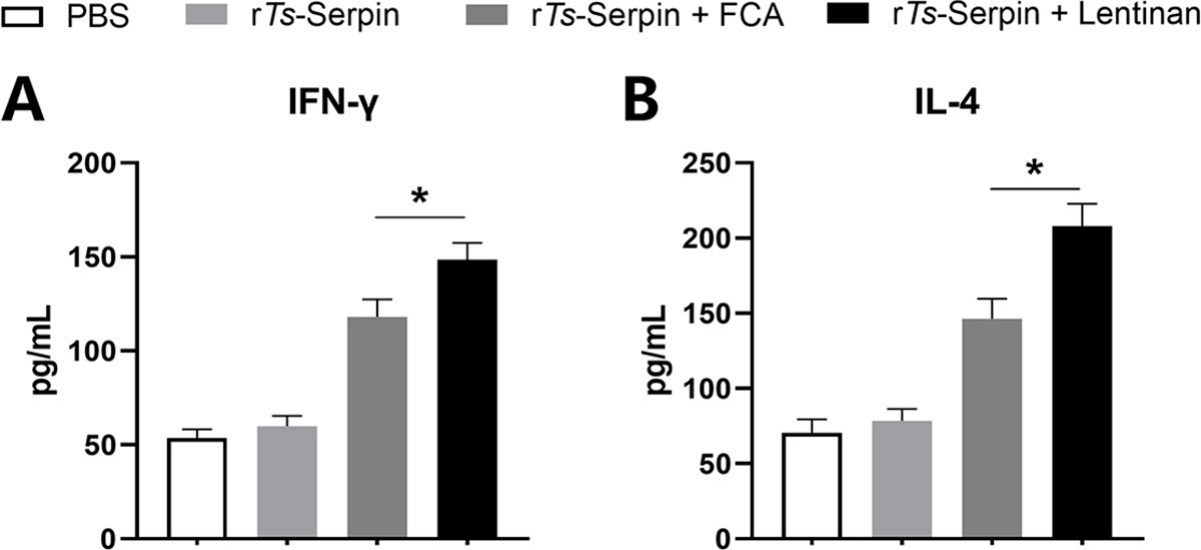
Analysis of cytokine production from CD4^+^ T cells. CD4^+^ T cells secreting IFN-γ (A) and IL-4 (B) production was measured by ELISA one week after the final immunization. The data are the mean ± SD of each group (n = 10) from three independent experiments. * p < 0.05 as indicated by the lines (one-way ANOVA with Tukey’s post-test).

Compared with r*Ts*-Serpin + FCA group, obviously higher levels of IFN-γ and IL-4 were found in the supernatants of r*Ts*-Serpin -stimulated CD4^+^ T cells from mice immunized with r*Ts*-Serpin + lentinan (Fig 3), suggesting that a stronger Th1/Th2-mixed response was induced by r*Ts*-Serpin + lentinan.

### Transcription factor NLRP3 in CD4^+^ T cells was associated with r*Ts*-Serpin + lentinan -induced protective efficacy

**Fig 4:**
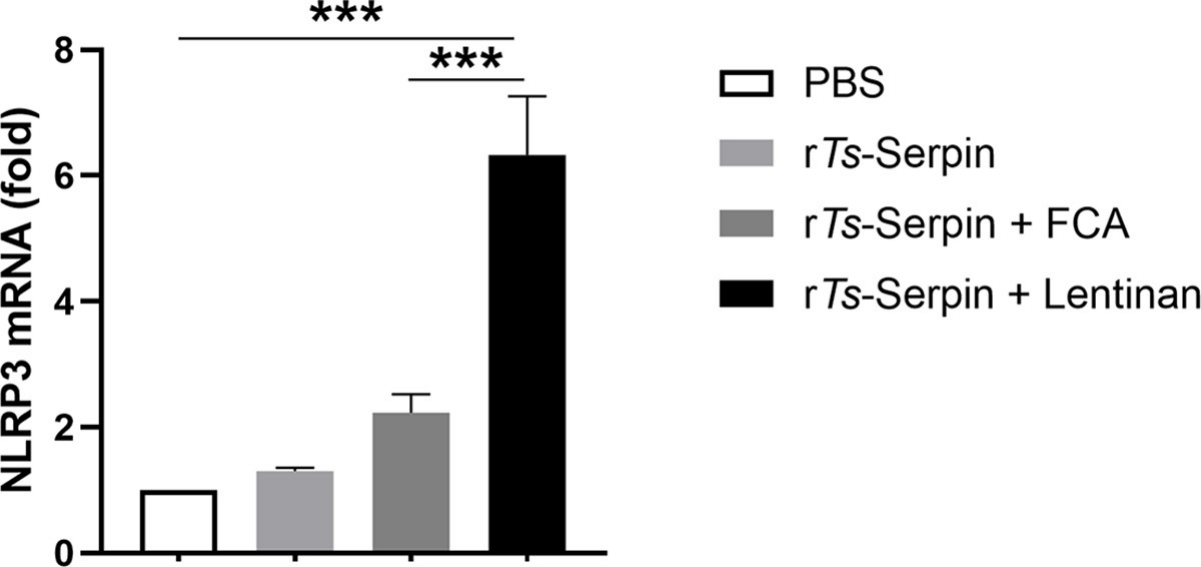
Expression of NLRP3 in CD4^+^ T cells. Results are relative to the expression at day 0. Results are the mean ± SD (n = 10) of three independent experiments. * p < 0.05, ** p < 0.01, *** p < 0.001 as indicated by line (one-way ANOVA with Tukey’s post-test).

**Fig 5:**
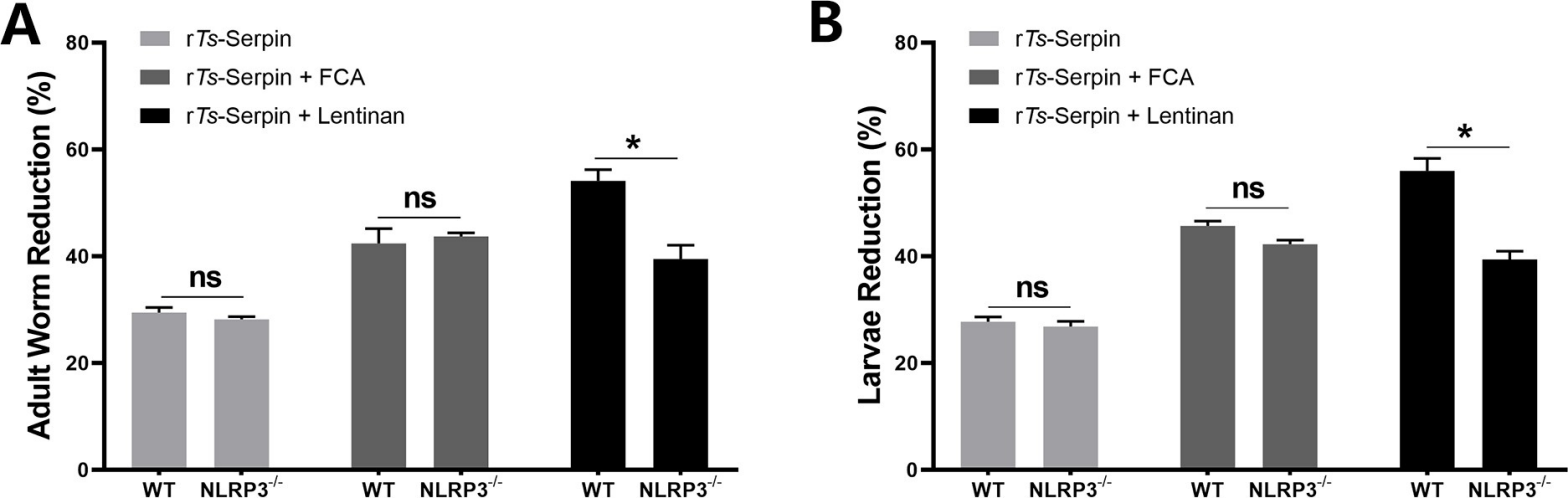
Protective immunity induced by immunizing WT and NLRP3^-/-^ mice with lentinan against *T*. *spiralis*. The adult worms (A) and muscle larvae (B) reduction rates were analyzed based on the the mean number of adult worms or the recovered muscle larvae per gram (LPG) of muscle from vaccinated groups compared with PBS group from immunized WT and NLRP3^-/-^ mice after challenge with 500 ML of *T*. *spiralis*. Results are expressed as the mean ± SD of 10 mice per group. The data shown are representative of three independent experiments. *p<0.05 vs the as indicated by the line (Tukey multiple comparison following ANOVA).

Recently, it has been reported that transcription factor NLRP3 in CD4+ T cells acts as a key transcription factor in Th2 immune response [18], which is associated with protective immunity to helminth infection. Thus, we investigated the role of NLRP3 in r*Ts*-Serpin + lentinan -induced immune protection against *T*. *spiralis*. First, we found that NLRP3 mRNA expression was significantly increased in CD4^+^ T cells isolated from mice immunized by r*Ts*-Serpin + lentinan compared with mice immunized by r*Ts*-Serpin + FCA (Fig 4), indicating that NLRP3 may play a role in lentinan -induced immunoprotection. Then, we analyzed the reduction rate of adult worm and muscle larvae burden in immunized WT and NLRP3^-/-^ mice. Our results demonstrated that in NLRP3^-/-^ mice immunized by r*Ts*-Serpin + lentinan displayed a significant lower reduction rate of parasite burden compared with that in WT mice (Fig 5), suggesting that NLRP3^-/-^ impaired the protective efficacy induce by r*Ts*-Serpin + lentinan.

### r*Ts*-Serpin+lentinan -induced Th2 protective immunity were decreased in NLRP3^-/-^ mice

**Fig 6:**
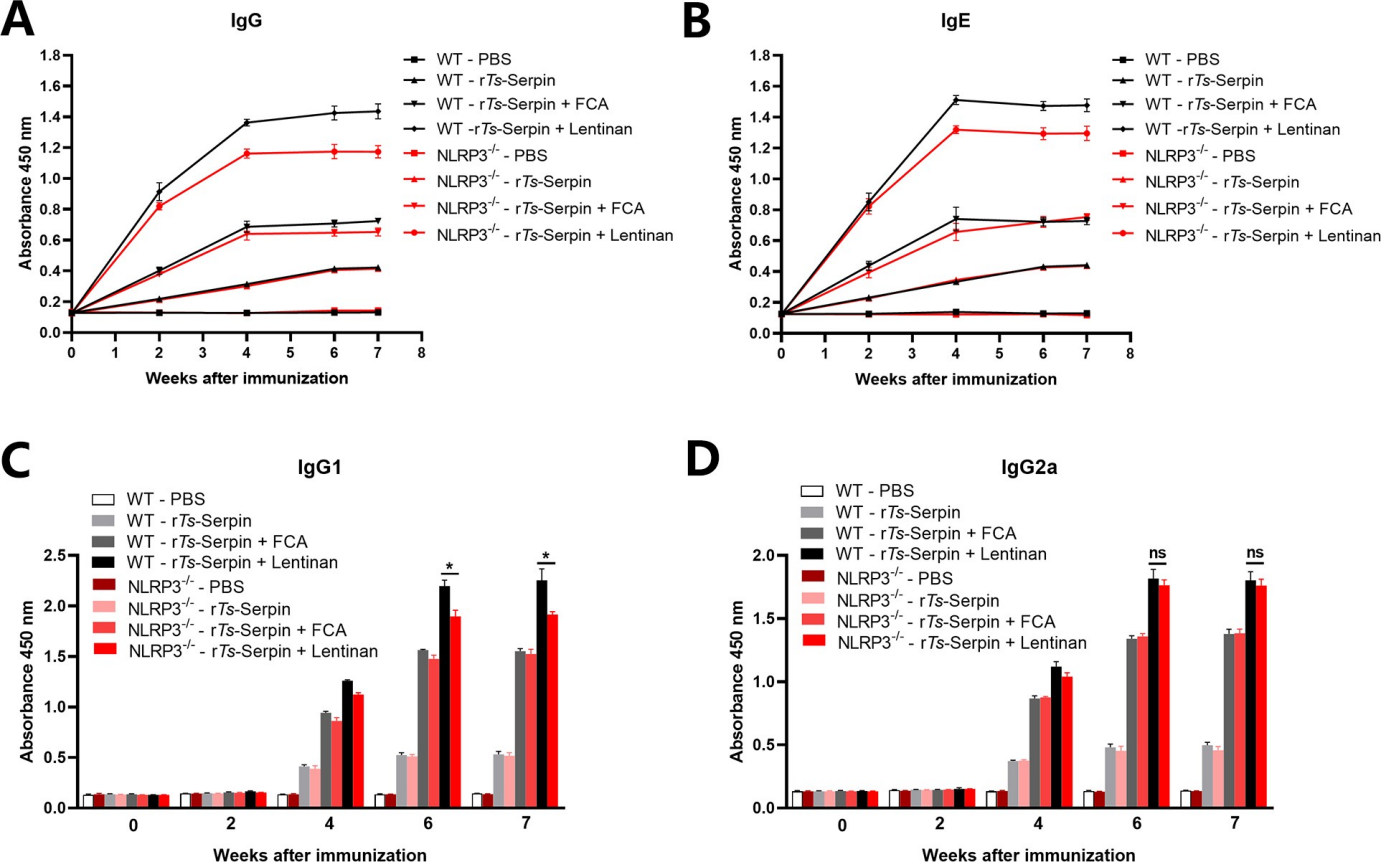
Analysis of humoral immune responses in WT and NLRP3^-/-^ mice. (A) The levels of anti-r*Ts*-serpin IgG in the sera of WT and NLRP3^-/-^ mice were measured by ELISA. (B) The levels of specific IgE r*Ts*-serpin in the sera of immunized mice were measured by ELISA. (C) The IgG1 subclass responses against r*Ts*-serpin in WT and NLRP3^-/-^ mice were detected at different time points. (D) The IgG2a subclass responses against r*Ts*-serpin in WT and NLRP3^-/-^ mice were detected at different time points. The values shown for each group are the mean + SD of the antibody levels (n = 10) from three individual experiments * p < 0.05 as indicated by the line (one-way ANOVA with Tukey’s post-test).

We observed that NLRP3^-/-^ mice immunized by r*Ts*-Serpin + lentinan had significant lower level of IgG and IgE compared with WT mice (Fig 6A and 6B). The level of IgG1 in NLRP3^-/-^ mice immunized by r*Ts*-Serpin+lentinan was significantly decreased compared with WT mice on 4 and 6 weeks (Fig 6B). However, little difference was found of the IgG2a level of WT and NLRP3^-/-^ mice (Fig 6C).

**Fig 7:**
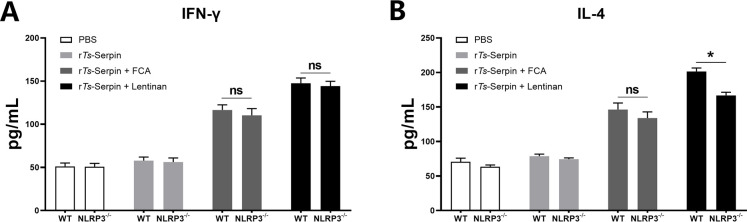
Analysis of cytokine production from WT and NLRP3^-/-^ CD4^+^ T cells. WT and NLRP3^-/-^ CD4^+^ T cells secreting IFN-γ (A) and IL-4 (B) production was measured by ELISA one week after the final immunization. The data are the mean ± SD of each group (n = 10) from three independent experiments. * p < 0.05 as indicated by the lines.

Moreover, we measured the levels of Th1 (IFN-γ) and Th2 (IL-4) cytokines secreted by CD4^+^ T cells. No significant difference of the level of IFN-γ between WT and NLRP3^-/-^ mice was observed (Fig 7A) meanwhile the IL-4 level of CD4^+^ T cells from NLRP3^-/-^ mice immunized by r*Ts*-Serpin+lentinan was significantly lower than that from WT mice (Fig 7B), indicating that NLRP3 was involved in the r*Ts*-Serpin + lentinan -induced Th2 response.

## Discussion

*Trichinella spp*., pathogenic agents of trichinellosis, is foodborne zoonotic nematodes cause huge economic burden to the livestock industry. Vaccination provides lifetime protection from the use of chemical antiparasitic drugs, reducing the emergence of drug-resistant *T*. *spiralis* and reducing consumer concerns about chemical residues in meat. The potential of new adjuvants for improving veterinary vaccines remains largely unexploited to trigger safe and long-lasting immunity in large animals, including livestock.

Natural polysaccharide has the characteristics of intrinsic immune regulation, biocompatibility, biodegradation, low toxicity and safety, and has attracted much attention in the preparation of vaccines and adjuvants [7, 10, 31]. Moreover, it has been proved that a variety of natural polysaccharides possess better immune promoting effects through enhancing the effects of humoral, cellular and mucosal immunities [32–35]. Lentinan is a polysaccharide and has been used previously as a biological response modifier and has been approved as an adjuvant for the treatment of gastric cancer and brought clinical benefits to cancer patients [36, 37]. And lentinan is a powerful adjuvant for favoring antiviral immunity [12]. β-glucan, as the main constituent of lentinan, enhances host response against bacterial infections [38]. Lentinan can reduce the burden of *Mesocestoides corti* [39] or *Schistosoma mansoni* [40], along with increased liver granulomas. In this paper, we first evaluated the prevention of lentinan against *T*. *spiralis* infection based on a vaccine. It has been reported that *T*. *spiralis* antigens are glycans with novel modifications, indicating that glycans are positioned to play important roles in parasitism, as well as immunity, in infection with this nematode [41]. Previously, we showed that r*Ts*-Serpin can elicit a significant protective immune response against *T*. *spiralis* [23]. In this study, we also confirmed the protective immunity of r*Ts*-Serpin against *T*. *spiralis* via inducing Th2 predominated Th1-/Th2-mixed type of immune response. The Th2-type CD4^+^ T cell response is characterized by the secretion of IL-4 providing B cell help and the preferential induction of IgG1 and IgE in mice [42]. It has been reported that the expulsion of *T*. *spiralis* is significantly delayed in the absence of IL-4, a Th2-related cytokine [17]. Therefore, the mechanism of lentinan -triggering Th2 protective immune response in the host is critical for the development of approaches to protect against *T*. *spiralis* infection.

Notably, NLRP3 is involved in adaptive immunity, which unlike ASC or Caspase-1, acts as a key transcription factor in Th2 immune response independent of inflammasome activation [18]. NLRP3 plays a role in the activities of the current adjuvants [21, 43]. In this paper, we observed that expression of transcription factor NLRP3 in CD4^+^ T cells was significantly increased in mice immunized by lentinan, based on vaccine, whereas expression of NLRP3 was not increased in CD4^+^ T cells from mice in vaccine group. We found that defect of NLRP3 impaired the ability of r*Ts*-Serpin + lentinan to protect against *T*. *spiralis* infection. And it has been reported that lentinan induces the activation of NLRP3 [44]. In addition, the level of IgE, IgG1 and IL-4, but not IgG2a and IFN-γ, in NLRP3^-/-^ mice immunized by r*Ts*-Serpin + lentinan was significantly decreased compared with WT mice, indicating NLRP3 participated in lentinan–induced protective efficacy through modulating Th2 immune response, which is associated with protective immunity to parasite infection. Evidence accumulated so far suggests the involvement of the NLRP3 inflammasome-independent pathways in the mechanisms of aluminum hydroxide-based adjuvants [45]. Certainly, this adjuvant employs more than one mechanism, since several different aspects of the immune system are affected, thus, further investigation is required to fully understand these pathways and desirable traits. NLRP3, after all, is one target of discovering more polysaccharide adjuvants for the more rational design of vaccine adjuvants.

Taken together, these findings demonstrated that lentinan significantly improved the protective efficacy of a vaccine against *T*. *spiralis* infection. And we identified the involvement of NLRP3 in lentinan–induced host defenses against *T*. *spiralis*. NLRP3 therefore represents an important target for adjuvant discovery and the control of *T*. *spiralis* infection.

## Supporting information

S1 Data Excel spreadsheet containing, in separate sheets, the underlying numerical data and statistical analysis for Figure panels 1A, 1B, 1C, 1D, 2A, 2B, 2C, 2D, 3A, 3B, 4, 5A, 5B, 6A, 6B, 6C, 6D, 7A, and 7B. (XLSX)
